# Herpetic Esophagitis: A Cause of Dysphagia in a Malnourished Patient

**DOI:** 10.7759/cureus.43858

**Published:** 2023-08-21

**Authors:** Bárbara Sousa, Joana Silva, Elsa Araújo, Raquel Costa, Andre Calheiros

**Affiliations:** 1 Internal Medicine, Unidade Local de Saúde do Alto Minho (ULSAM), Ponte de Lima, PRT

**Keywords:** immunocompetent patients, herpes simplex virus, herpetic esophagitis, esophageal stenosis, dysphagia

## Abstract

Herpetic esophagitis (HE) is an esophageal infection caused by herpes simplex virus (HSV). Although less common, it can occasionally affect immunocompetent hosts. It can manifest as odynophagia and/or dysphagia and should lead to an investigation by upper digestive endoscopy with a biopsy.

The authors report a case of a 65-year-old man with a past medical history relevant for schizophrenia, oligophrenia, and malnutrition, and no other history or evidence of immunosuppression, who presented with severe dysphagia over weeks and recent episodes of food aspiration with consequent pneumonia. An upper gastrointestinal endoscopy was performed, revealing severe stenosis at the level of the gastroesophageal junction with scar tissue, not transposable with the endoscope. The biopsy led to the diagnosis of hepatic esophagitis. Despite the immunocompetent status (excepting only the risk factor malnutrition) and treatment with acyclovir, with initial clinical improvement, the patient died a few weeks after diagnosis after multiple respiratory complications such as nosocomial infection.

This case highlights that herpetic esophagitis is sometimes observed in immunocompetent hosts. HE has a self-limited course, with severe complications more frequent in immunosuppressed patients. However, it is also important to suspect this condition in immunocompetent patients and look for risk factors, given the potential morbidity this disease entails. In this group of patients, the presence of predisposing factors and associated comorbidities, such as malnutrition, alcohol consumption, or use of corticosteroids, have been associated with the development of viral esophagitis (including HE).

HE remains a clinical challenge, especially in patients with risk factors for immunosuppression, such as malnutrition, as in the reported case.

## Introduction

Herpetic esophagitis (HE) is defined as an esophageal infection caused by herpes simplex virus (HSV) and mainly affects immunocompromised patients. Although less frequent, there are case reports of herpetic esophagitis in immunocompetent patients [1-2]. In these patients, this infection is frequently a self-limited disorder. Treatment with acyclovir can accelerate their resolution and improve symptoms [3-4].

HE usually presents with odynophagia and/or dysphagia, and its diagnosis is based on endoscopy and histopathology examination [3]. Severe symptoms and complications (like esophageal stenosis, perforation, or bleeding) are much more frequent in immunosuppressed patients.

## Case presentation

We report a case of a 65-year-old man, with a past medical history relevant for schizophrenia, oligophrenia, and malnutrition, on chronic anti-psychotic medication, living in a nursing home, and dependent on self-care in the context of his psychiatric illness, who presented with worsening of dysphagia within two weeks, with no other complaints or any visible oropharyngeal lesions and no previous history of herpes infection. He was admitted to the emergency department due to severe dysphagia and recent episodes of food aspiration with associated shortness of breath. The laboratory data were significant only for an elevation of inflammatory markers and were otherwise unremarkable. This patient had no evidence of acquired immunodeficiency syndrome, hematologic malignancies, solid tumors, and no history of transplants or undergoing treatment with immunosuppressive drugs. He was diagnosed with pneumonia and hypoxemic respiratory failure, probably secondary to aspiration, and started on antibiotic therapy and supplemental oxygen with hospitalization.

Despite his cognitive limitations due to his psychiatric illness, which could make it difficult to interpret multiple subjective complaints, the picture of severe dysphagia was evident. Due to dysphagia, he underwent upper gastrointestinal endoscopy, revealing stenosis at the level of the gastroesophageal junction with scar tissue, not transposable with the endoscope. A biopsy of the lesion was performed and a nasogastric tube for feeding was placed.

**Figure 1:**
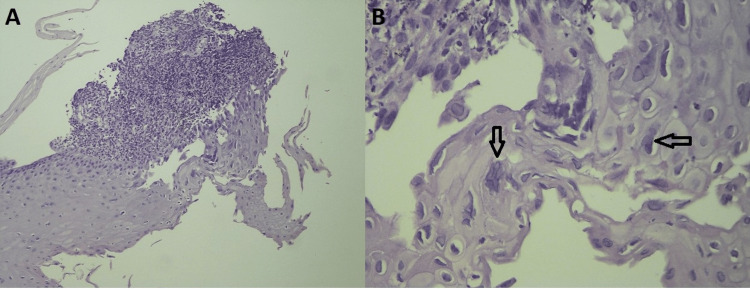
Esophageal biopsy with typical histologic finding for herpes simplex virus (HSV) infection A - Low magnification view of esophageal biopsy showing reactive squamous mucosa at the base and an acute inflammatory cellular infiltrate surrounding virally infected cells superficially (H&E, 100x); B - High magnification view of viral cytopathic effects showing multinucleation (arrows) and nuclear chromatin margination, giving the nuclei a ground-glass appearance (H&E, 400x) Contributed by the Department of Pathological Anatomy of Unidade Local de Saúde do Alto Minho

**Figure 2:**
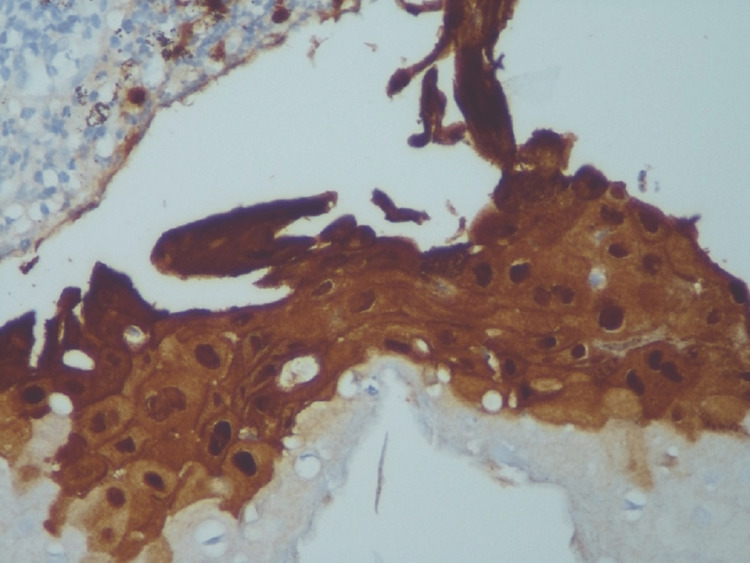
HSV1 immunohistochemistry staining virally infected cells HSV1: herpes simplex virus type 1 Contributed by the Department of Pathological Anatomy of Unidade Local de Saúde do Alto Minho

The result of the biopsy showed fragments of mucosa with areas of mononuclear cell infiltration and epithelial changes with viral cytopathic effects (Figure 1) with a negative study for malignancy or other causes of esophagitis. Immunohistochemistry study stained virus-infected epithelial cells (Figure 2). The polymerase chain reaction was positive for herpes simplex virus type 1 (HSV-1). The cause of the stenosis was undoubtedly herpetic infection by excluding all causes as well as clinical and histological evidence compatible with the diagnosis.

In order to exclude a neoplastic cause for the stenosis, in addition to the negative study for malignancy in the biopsy of multiple fragments studied, serum tumor markers were also requested (such as CEA and CA 19.9) with negative results. The study was performed by computed tomography (CT) of the neck and thorax, without evidence of another cause of stenosis and no evidence of malignancy. Analytically, with mild hypoalbuminemia (albumin 3.0 g/dL with reference values ​​between 3.5 and 5.5 g/dL) suitable in the context of malnutrition of patients, and normal immunoglobulin dosage and protein electrophoresis, negative HIV serology with serological tests for the detection of IgM and IgG antibodies to HSV-1 was positive. The antinuclear antibody (ANA) assay was also negative, and there were no other clinical findings that would suggest autoimmune diseases such as scleroderma.

Therefore, after the complete study, the patient was diagnosed with herpetic esophagitis and was started on intravenous acyclovir with clinical improvement and tolerance to oral feeding, as well as resolution of pneumonia and respiratory failure. It was also observed by the hospital nutrition team, with adjustment of the nutritional intake. The patient was discharged on oral acyclovir, making up a total of 14 days of treatment, and was scheduled for a follow-up consultation.

Despite the clinical improvement and apparent positive response to therapy, the patient continued to have several complications from pulmonary infections without improvement after adequate therapy and support, and he died a few weeks after diagnosis in the course of nosocomial infection with multiorgan failure.

## Discussion

Severe esophageal stenosis, due to progressive esophagitis, such as the one presented, can lead to multiple differential diagnoses. Although the infection's causes are less likely to be the etiology, there are a few case reports of infections such as herpetic esophagitis in the literature [4-6]. Herpetic esophagitis occurs mainly in immunocompromised patients, such as acquired immunodeficiency syndrome, hematological neoplasms, solid tumors, transplant patients, or patients undergoing treatment with immunosuppressive drugs [7].

Some authors argue that treatment differs according to the immunological status of the patient and clinical severity [1,8-9]. Although spontaneous resolution usually occurs after one to two weeks, patients may improve more quickly if treated with a short course of acyclovir, which may be beneficial in accelerating the resolution of the symptoms, with minimal or no toxicity [1,6,10]. Special care should also be taken with patients with severe symptoms, such as dysphagia, who may require hospitalization for parenteral antiviral therapy and feeding.

In 2019, Hoversten et al. analyzed patients with herpes simplex esophagitis (HSE) from the Mayo Clinic pathology database from 2006 to 2016 (N=46). The aim was to study the clinical course of HSE based on the degree of immunocompetence and the presence of underlying esophageal disease. The disease course seems to be self-limited for all patients; however, it can impact immunocompetent patients or those without underlying esophageal disease, which may have resulted in delayed diagnosis and treatment in these patient populations [11].

Therefore, it is important to also suspect herpetic esophagitis in immunocompetent patients and look for risk factors, given the potential morbidity this disease entails. In this group of patients, the presence of predisposing factors and associated comorbidities, such as malnutrition, alcohol consumption, or the use of corticosteroids, have been associated with the development of viral esophagitis (including HSV) [12].

Despite this patient's cognitive limitations due to his psychiatric illness, which could make it difficult to interpret multiple subjective complaints, the picture of severe dysphagia was evident. Dysphagia was caused by severe esophageal stenosis and the cause of the stenosis was undoubtedly herpetic infection by excluding all causes as well as clinical and histological evidence compatible with the diagnosis.

Although malnutrition may be a risk factor for immunosuppression, there was no other evidence of an immunosuppression state in this patient. It is also noted that serum immunoglobulins were normal, including positive antibodies for HSV, showing some immunological competence.

So, herpetic esophagitis can be the cause of dysphagia in patients, regardless of their immunocompetence status. In this clinical case, the authors highlight the various aspects that have become a clinical challenge, such as the clinical presentation with severe dysphagia that led to the survey and exclusion of other causes, such as neoplasia, the patient's nutritional status, and culminating in the unfortunate death due to aspiration-related complications and nosocomial infection.

## Conclusions

This case suggests that herpetic esophagitis should be considered regardless of the patient's immune status. Although herpetic esophagitis is usually self-limited, with symptoms resolution over the first two weeks, more severe complications can arise, especially in immunocompromised patients or with risk factors for immunosuppression, such as malnutrition, as in the case reported.

Therefore, this clinical case highlights the diagnosis of herpetic esophagitis in the management of patients with severe dysphagia, highlighting the importance of HSV as a pathogen. Also noteworthy is the nutritional assessment of patients and the need to treat malnutrition, especially in patients with risk factors, such as psychiatric illnesses, in improving the prevention and treatment of infections.

## References

[1] McBane RD, Gross JB Jr (1991). Herpes esophagitis: clinical syndrome, endoscopic appearance, and diagnosis in 23 patients. Gastrointest Endosc.

[2] McDonald GB, Sharma P, Hackman RC (1985). Esophageal infections in immunosuppressed patients after marrow transplantation. Gastroenterology.

[3] Ramanathan J, Rammouni M, Baran J Jr, Khatib R (2000). Herpes simplex virus esophagitis in the immunocompetent host: an overview. Am J Gastroenterol.

[4] Canalejo E, García Durán F, Cabello N, García Martínez J (2010). Herpes esophagitis in healthy adults and adolescents: report of 3 cases and review of the literature. Medicine (Baltimore).

[5] Kadayakkara DK, Candelaria A, Kwak YE, Loeser C (2016). Herpes simplex virus-2 esophagitis in a young immunocompetent adult. Case Rep Gastrointest Med.

[6] Diezma-Martín AM, Gigante-Miravalles E, Castro Limo JD, Quimbayo Arcila CA, Puche Paniagua JJ (2020). Herpetic esophagitis in immunocompentent host: cases report. BMC Infect Dis.

[7] Ahuja NK, Clarke JO (2016). Evaluation and management of infectious esophagitis in immunocompromised and immunocompetent individuals. Curr Treat Options Gastroenterol.

[8] Becker K, Lübke HJ, Borchard F, Häussinger D (1996). Inflammatory esophageal diseases caused by herpes simplex virus infections--overview and report of 15 personal cases [Article in German]. Z Gastroenterol.

[9] Asano N, Kano Y, Takarada C (2021). Glossitis and esophagitis from herpes simplex virus type 1 infection. CMAJ.

[10] Wang HW, Kuo CJ, Lin WR (2016). Clinical characteristics and manifestation of herpes esophagitis: one single-center experience in Taiwan. Medicine (Baltimore).

[11] Hoversten P, Kamboj AK, Wu TT, Katzka DA (2019). Variations in the clinical course of patients with herpes simplex virus esophagitis based on immunocompetence and presence of underlying esophageal disease. Dig Dis Sci.

[12] Hoversten P, Kamboj AK, Katzka DA (2018). Infections of the esophagus: an update on risk factors, diagnosis, and management. Dis Esophagus.

